# Neuroprotective effect of *Aronia melanocarpa* extract against glutamate-induced oxidative stress in HT22 cells

**DOI:** 10.1186/s12906-017-1716-1

**Published:** 2017-04-11

**Authors:** Hyeon Yong Lee, Jin Bae Weon, Gahee Ryu, Woo Seung Yang, Nam Young Kim, Myong Ki Kim, Choong Je Ma

**Affiliations:** 1 Department of Food Science and Engineering, Seowon University, Cheongju, 361-742 Republic of Korea; 2 Future Food Research Center, 377-3 Musimsero, Seowon-gu, Cheongju, Chungbuk 28674 Republic of Korea; 3 Department of Medical Biomaterials Engineering, College of Biomedical Science, Kangwon National University, Hyoja-2 Dong, Chuncheon, 200-701 Republic of Korea; 4 Institute of Bioscience and Biotechnology, Kangwon National University, Chuncheon, 200-701 Republic of Korea

**Keywords:** *Aronia melanocarpa*, Neuroprotective effect, Glutamate, Antioxidant effect

## Abstract

**Background:**

Glutamate (an endogenous excitatory neurotransmitter) at high concentrations contributes to the development of neurodegenerative diseases. *Aronia melanocarpa* (*A. melanocarpa*) berries contain anthocyanins and have high antioxidant activities. In this study, we evaluated whether *A. melanocarpa* berries could protect neuronal cells against glutamate-induced oxidative stress.

**Method:**

*A. melanocarpa* berries exerted a protective effect against cytotoxicity in HT22 mouse hippocampal cells by MTT assay. We evaluated oxidative stress parameters including ROS level, intracellular Ca^2+^ level, glutathione level and antioxidant enzyme activity in HT22 cells to elucidate the mechanism of its neuroprotective effect.

**Results:**

*A. melanocarpa* berries decreased glutamate-induced death of HT22 cells. In addition, *A. melanocarpa* berries reduced ROS and intracellular Ca^2+^ levels. Glutathione level, antioxidant enzymes, glutathione reductase and glutathione peroxide activities and mitochondrial membrane potential were also increased in HT22 cells.

**Conclusion:**

These results suggested that *A. melanocarpa* berries protected HT22 cells by exerting an antioxidant effect.

## Background

Neurodegenerative disorders, including Alzheimer’s disease (AD), Parkinson’s disease and Huntington’s disease are characterized by loss of neuronal function and memory and cognitive impairment [1]. AD is the most common neurodegenerative disorder [2]. Oxidative stress, including lipid peroxidation, free radical formation, protein oxidation and DNA oxidation, in the central nervous system (CNS) can lead to cell death and contributes to the pathogenesis of various neurodegenerative disorders [3, 4].

Glutamate is an excitatory neurotransmitter that plays a role in learning and memory, and contributes to excitotoxicity in neuronal cells [5]. Glutamate is one of the most important mechanisms known to trigger and neuroinflammation and neuronal cell death in CNS disorders. Glutamate-induced excitotoxicity may contribute to the neuronal injury in neurodegenerative diseases such as motor neuron disease and Alzheimer’s disease by neuronal injury as anoxia and reperfusion [6].

Excessive glutamate concentration can induce oxidative stress by increasing the production of reactive oxygen species (ROS) and intracellular calcium (Ca^2+^) levels. Glutamate excitotoxicity also results in mitochondrial dysfunction and depletion of antioxidant defense systems, including glutathione (GSH), glutathione peroxidase (GPx) and glutathione reductase (GR) by inhibiting cystine uptake [7–9].

*Aronia melanocarpa* (black chokeberry: *A. melanocarpa*), a member of the Rosaceae family, is a fruit with a high content of polyphenols, including anthocyanins (cyanidin glycosides), flavanols, flavonoids (quercetin glycosides), chlorogenic acids, triterpenes, and fibers and caffeic acid derivatives [10]. *A. melanocarpa* has shown high antioxidant activity, as well as hepatoprotective, gastroprotective and anti-inflammatory effects. Recent studies showed that *A. melanocarpa* prevents obesity in C57BL/6 J mice and reduces systolic and diastolic blood pressure [11, 12]. *A. melanocarpa* also protects against female skeleton damage due to chronic exposure to Cd [13]. Cyanidin-3-O-galactoside is a major compound in *A. melanocarpa,* has considerable antioxidant activity and protects against endothelial dysfunction and vascular failure induced by peroxynitrite [14, 15].

Trolox is water-soluble derivative of vitamin E and antioxidant to reduce oxidative stress. it play as positive control in this study.

The aim of present study was to investigate the neuroprotective effect of *Aronia melanocarpa* and the possible underlying mechanism against glutamate-induced death of HT22 cells.

## Methods

### Plant material and extraction

*A. melanocarpa* berries (Danyang, Chungcheongbuk-do, Korea) from a 14-year-old plant were collected and authenticated by Dr. Young Bae Seo, a professor of the College of Oriental Medicine, Daejeon University, Korea. A voucher specimen (CJ200M) has been deposited in the natural products laboratory, the Kangwon National University. *A. melanocarpa* extract was obtained from the Future Food Research Center (Cheng Ju, Korea).

*A. melanocarpa* berries were pulverized using a blender after freeze-drying for 3 days (PVTFA 10AT, ILSHIN BioBase, Dongducheon, Korea). Powdered *A. melanocarpa* berries were extracted in 70% ethanol (100 g/1 L) at room temperature by maceration and were filtered through a vacuum filter. The extract was concentrated by evaporation (EYELA N-1000, Tokyo Rikakikai Co, Tokyo, Japan) and then freeze-dried for 3 days.

### Cell viability assay

Cell viability was investigated by MTT assay using a method described previously [16]. HT22 cells were seed at a density of 6.7 × 10^4^/well in 48-well plates and incubated at 37 °C in 5% CO_2_. After incubation for 24 h, 10 and 100 μg/ml of extract, 1 and 10 μg/ml of cyanidin-3-O-galactoside, trolox (positive control, 50 μM) and glutamate were added. Then, cells were incubated for 3 h with dimethyl thiazolyl diphenyl tetrazolium salt (MTT) (1 mg/ml) solution, and dimethyl sulfoxide (DMSO) was added to dissolve MTT-formazan crystals. The optical density at 570 nm was measured using an ELISA reader.

### ROS measurement

2`7`-Dichlorofluorescein diacetate (DCF-DA) was used for measurement of ROS levels. HT22 cells (6.7 × 10^4^/well in 48-well plates) were treated with 10 and 100 μg/ml of extract, 1 and 10 μg/ml of cyanidin-3-O-galactoside, trolox (positive control, 50 μM) and 2 mM glutamate for 8 h. Then, cells were washed with PBS and incubated in 10 μM DCF-DA in Dulbecco’s modified Eagle’s medium (DMEM) without phenol red for 30 min. Cells were washed twice with phosphate buffer saline (PBS), 1% Triton X-100 added, and incubated for 10 min at 37 °C. Fluorescence was measured at an excitation wavelength of 490 nm and emission wavelength of 525 nm.

### Calcium (Ca^2+^) measurement

Intracellular Ca^2+^ levels were measured using the Fura-2 AM. HT22 cells were plated in 48-well plates and incubated for 24 h at 37 °C and 5% CO_2_. After incubation, 10 and 100 μg/ml of extract, 1 and 10 μg/ml of cyanidin-3-O-galactoside, Trolox (positive control, 50 μM) and 2 mM glutamate were treated for 3 h, and then 2 μM Fura-AM was added to each well. Cells were then washed three times with HEPES buffer saline. Ca^2+^ levels were determined by measuring fluorescence intensity at an excitation wavelength of 340 and 380 nm and emission wavelength of 500 nm.

### Mitochondrial membrane potential (ΔΨ) measurement

Mitochondrial membrane potential (ΔΨ) change was determined by monitoring the accumulation of the fluorescent dye, rhodamine 123 (Rho123). HT22 cells were treated with 2 mM glutamate, 10 and 100 μg/ml of *A. melanocarpa* extract and 1 and 10 μg/ml of cyanidin-3-O-galactoside. HT22 cells were then stained with Rho123 for 15 min at 37 °C and washed. The Rho123 concentration was measured by spectrofluorometry at an excitation wavelength of 488 nm and emission wavelength of 520 nm.

### GSH measurement

Total GSH was measured by an enzymatic cycling method based on the reduction of 5′,5′-dithiobis 2-nitrobenzoic acid (DTNB) with GSH reductase and nicotinamide adenine dinucleotide phosphate (NADPH). Cells were treated with extract, cyanidin-3-O-galactoside and glutamate for 8 h and washed with 0.2 M phosphate buffer (pH 7.4). Cells were lysed with sulfosalicylic acid and centrifuged at 3000 g for 30 min at 4 °C to collect supernatants. Supernatants were mixed with 5 units/mL glutathione disulfide reductase, 0.3 mM NADPH and 0.5 mM DTNB. The reaction absorbance at 412 nm was measured within 15 min.

### Antioxidant enzyme, glutathione reductase and glutathione peroxidase assays

HT22 cells were treated with extract, cyanidin-3-O-galactoside and glutamate for 8 h. Cells were washed with 0.2 M phosphate buffer (pH 7.4) and lysed with sulfosalicylic acid. After centrifugation (3000 g for 30 min at 4 °C), supernatants were collected. Glutathione reductase (GR) was measured by monitoring the reduction of oxidized GSH (GSSG) in the presence of NADPH. Glutathione peroxidase activity was determined by quantifying the rate of oxidation of GSH to GSSG. The decrease in absorbance at 340 nm was measured using a spectrophotometer.

### Statistics

All data were expressed as means ± S.D. Significant differences were analyzed by one-way analysis of variance (ANOVA) followed by Tukey’s post hoc test. Statistical significance was set at *P* < 0.05, 0.01 and 0.001.

## Results

### Neuroprotective effect of *A. melanocarpa* on glutamate-induced cell death in HT22 cells

**Fig. 1:**
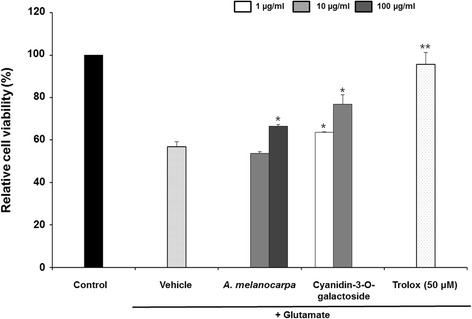
Effect of *A. melanocarpa* extract (10 and 100 μg/ml) and cyanidin-3-O-galactoside (1 and 10 μg/ml) on glutamate-induced death of HT22 cells. Data are means ± S.D. *p < 0.05 versus the glutamate-treated group

We evaluated the neuroprotective effects of *A. melanocarpa* extract in HT22 cells (Fig. 1). *A. melanocarpa* extract exhibited significant neuroprotective effects by reducing glutamate-induced cell death to 16.81 ± 35.38% (relative protection at 100 μg/ml). Cyanidin-3-O-galactoside also significantly protected HT22 cells against glutamate-induced neurotoxicity, with relative protection of 35.38 ± 12.43% at 10 μg/ml.

### ROS production

**Fig. 2:**
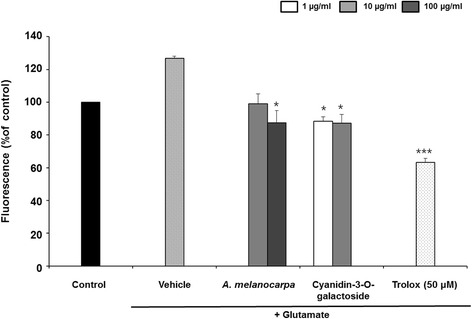
Effect of *A. melanocarpa* extract (10 and 100 μg/ml) and cyanidin-3-O-galactoside (1 and 10 μg/ml) on ROS production in HT22 cells. Data are means ± S.D. *p < 0.05 versus the glutamate-treated group

Inhibition of ROS production by *A. melanocarpa* extract was evaluated using 2`7`-dichlorofluorescein diacetate (Fig. 2). Glutamate treatment increased the ROS level in HT22 cells compared to the control (126.7 ± 1.32%). *A. melanocarpa* extract at 100 μg/ml significantly decreased glutamate-induced ROS production to 87.32 ± 7.50% (*p* < 0.05). Cyanidin-3-O-galactoside at 10 μg/ml also decreased ROS production to 87.23 ± 5.30%.

### Intracellular Ca^2+^ production

**Fig. 3:**
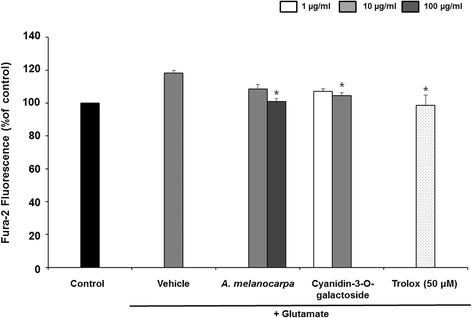
Effect of *A. melanocarpa* extract (10 and 100 μg/ml) and cyanidin-3-O-galactoside (1 and 10 μg/ml) on Ca^2+^ influx in HT22 cells. Data are means ± S.D. *p < 0.05 versus the glutamate-treated group

High concentrations of glutamate lead to intercellular Ca^2+^ accumulation. We investigated intracellular Ca^2+^ levels using Fura-AM to determine the effect of *A. melanocarpa* extract in HT22 cells (Fig. 3). *A. melanocarpa* extract at 100 μg/ml significantly decreased the intracellular Ca^2+^ concentration in HT22 cells compared to glutamate-treated cells (100.81 ± 1.89% (*p* < 0.05) at 100 μg/ml). Cyanidin-3-O-galactoside at 10 μg/ml also significantly decreased intracellular Ca^2+^ levels to 104.37 ± 1.80%.

### Mitochondrial membrane potential (ΔΨ)

**Fig. 4:**
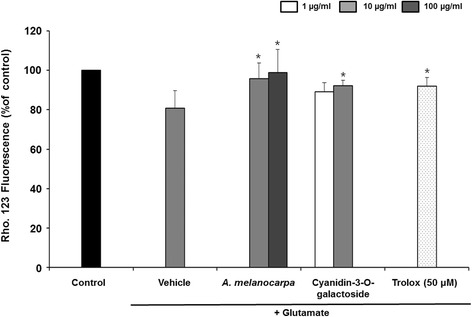
Effect of *A. melanocarpa* extract (10 and 100 μg/ml) and cyanidin-3-O-galactoside (1 and 10 μg/ml) on glutamate-induced disruption of mitochondrial membrane potential. Data are means ± S.D. *p < 0.05 versus the glutamate-treated group

We investigated the effect of *A. melanocarpa* extract on mitochondrial membrane potential (ΔΨ) in HT22 cells using Rho123 dye (Fig. 4). Glutamate-treated HT22 cells exhibited a decreased mitochondrial membrane potential to 80.94 ± 8.70%. *A. melanocarpa* extract at 100 μg/ml significantly increased the mitochondrial membrane potential to 98.84 ± 11.90% of the control. In addition, Cyanidin-3-O-galactoside improved the mitochondrial membrane potential decreased by glutamate (92.25 ± 2.62% at 10 μg/ml).

### GSH level and GR and GPx activity

**Fig. 5:**
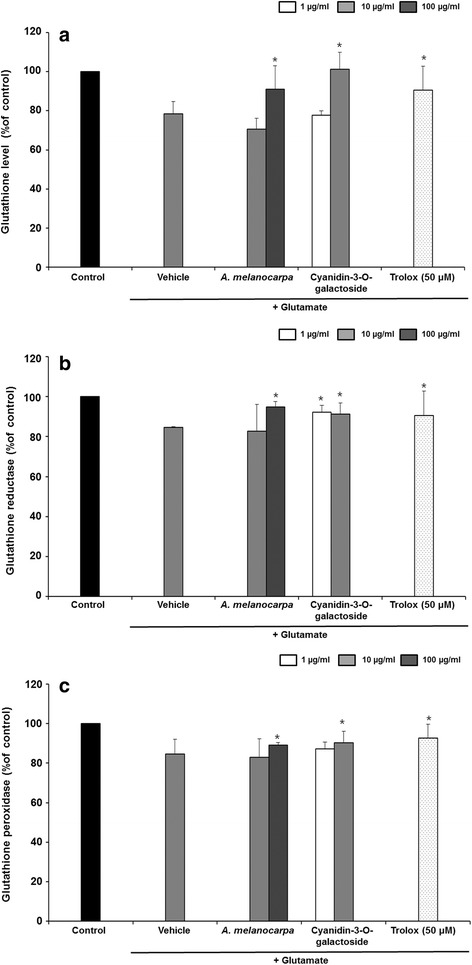
Glutathione (GSH) **a**, glutathione reductase (GR) **b** and glutathione peroxidase (GPx) **c** levels in HT22 cells. Data are means ± S.D. *p < 0.05 versus the glutamate-treated group

We evaluated the effect of *A. melanocarpa* extract on GSH level, and GR and GPx activity in HT22 cells (Fig. 5). *A. melanocarpa* extract increased the GSH level. Exposure to glutamate increased the GSH level by 78.56 ± 6.26%, GR activity by 84.79 ± 0.29% and GPx activity by 89.04 ± 1.36%, whereas *A. melanocarpa* extract prevented the glutamate-induced depletion of GSH level (90.92 ± 12.19% at 100 μg/mL), GR activity (94.90 ± 2.68% at 100 μg/mL) and GPx activity (89.05 ± 1.36% at 100 μg/mL). Cyanidin-3-O-galactoside also significantly increased the GSH level, GR activity and GPx activity.

## Discussion

This study demonstrated that *A. melanocarpa* extract protected mouse hippocampal HT22neuronal cells against glutamate-induced death.

High levels of glutamate excitotoxicity can lead to neuronal cell death by oxidative stress. It is also involved in intercellular Ca^2+^ influx and reactive oxygen species (ROS) generation via NMDA receptor [17]. ROS, including hydroxyl radical (OH^−^), superoxide anion (O_2_^−^) and hydrogen peroxide (H_2_O_2_), are generated in cells and lead to death due to DNA damage, protein oxidation, and lipid peroxidation [18]. Intercellular Ca^2+^ influx contributes to excessive ROS production and causes depolarization of the mitochondrial membrane. GSH is an important antioxidant involved in nutrient metabolism, DNA synthesis, signal transduction and cell proliferation in the CNS. GR and GPx are critical enzymes for the production of GSH. A high concentration of glutamate was involved in depletion of GSH by inhibiting cysteine uptake into cells [19]. Depletion of GSH or antioxidant enzymes, such as GR and GPx, leads to neuronal cell death [20]. Mitochondria play an important role in neuronal cell death. Mitochondrial dysfunction results in ROS production and cell apoptosis and is indicated by loss of the mitochondrial membrane potential [21]. A high glutamate level also induced a decrease in the mitochondrial membrane potential in cells in the CNS.

HT22 cells, an immortalized mouse hippocampal cell line, are used in vitro for mechanistic studies related to glutamate-induced cell death by oxidative stress [22].

Our results showed that *A. melanocarpa* extract protected HT22 cells against glutamate-induced cell death by inhibiting ROS generation and Ca^2+^ influx. *A. melanocarpa* extract also restored GSH, GPx and GR activity and increased the mitochondrial membrane potential. Therefore, the neuroprotective effect of *A. melanocarpa* extract against glutamate-induced cell death was likely mediated through attenuation of oxidative stress.

Cyanidin-3-O-galactoside is a major compound in *A. melanocarpa* extract. A previous study demonstrated that cyanidin-3-O-galactoside exerted an antioxidant effect and a cognitive effect on spatial memory, and regulates hippocampal ERK expression in senescence-accelerated mice [23]. Thus, cyanidin-3-O-galactoside may be involved in the neuroprotective effect of *A. melanocarpa* extract.

## Conclusion

In conclusion, *A. melanocarpa* extract protected neuronal cells against glutamate-induced death due to its antioxidant activity. Therefore, *A. melanocarpa* extract may have therapeutic potential for neurodegenerative diseases, such as Alzheimer’s disease.
